# Is SWEEPS better than PUI in reducing intracanal bacteria and inflammation in cases of apical periodontitis?

**DOI:** 10.1007/s10103-024-04117-9

**Published:** 2024-07-16

**Authors:** Yelda Erdem Hepsenoglu, Seyda Ersahan, Erhan Erkan, Mustafa Gundogar, Fatih Ozcelik

**Affiliations:** 1 Department of Endodontics, Faculty of Dentistry, Istanbul Medipol University, Birlik Mah. Bahçeler Cad. No: 5 Esenler, Istanbul, Turkey; 2 Department of Medical Biochemistry Department, Health Sciences University, Sisli Hamidiye Etfal Training and Research Hospital, Istanbul, Turkey

**Keywords:** Apical periodontitis, Irrigation activation, SWEEPS, Passive ultrasonic system, PCR, Biomarker, IL-1β

## Abstract

To evaluate the efficacy of SWEEPS mode of the Er: YAG laser(SL) and passive ultrasonic irrigation(PUI) in the eradication of microorganisms and in the inflammation detection by IL-1β. Thirty patients with chronic apical periodontitis(AP) were allocated into two groups: Group SL–SWEEPS laser activated irrigation(*n* = 15) and Group PUI–passive ultrasonic irrigation(*n* = 15). Bacteriological samples were taken before(S1) and after chemomechanical preparation(S2), and then after final irrigation activation(S3). The levels of total bacteria and *Streptococci* were measured by means of PCR. Blood samples were collected before and 3rd day after treatment. Enzyme-linked immunosorbent assay was used to measure the levels of IL-1β. The bacterial reduction showed no differences between groups after chemo-mechanical treatment and after irrigant activation(*p* = 0.590). Post-treatment IL-1β levels were lower than pretreatment levels in both groups(*p* < 0.001). SL or PUI application in addition to chemomechanical preparation has similar effects on total bacterial level and inflammation detected by IL-1β in patients with AP.

## Introduction

Apical periodontitis (AP) is a prevalent infectious disease worldwide and these lesions represent an inflammatory/immune pathosis affecting the periapical tissue including the surrounding bone. This periapical process is primarily initiated by bacterial infection in the necrotic pulp. Its persistence, progression to chronic lesions, and destruction of bone structures are a consequence of the inability of host defense mechanisms to eradicate infection [1]. Bacterial products, host immune cells and biologically active factors called locally produced cytokines (such as IL-1β, IL-6, TNF-α) have been reported to play an important role in the pathogenesis of AP [2]. Interleukin-1 beta (IL-1β) has been considered as a major potent mediator of bone resorption and implicated in the development of human periapical lesions. No clinical investigations were undertaken to correlate the level of IL-1β, a pro-inflammatory cytokine, with bacterial load and lesion size in AP.

The successful treatment of AP depends on the maximum decrease in microorganisms and their by-products in root canals [3]. Chemomechanical preparation of the root canal alone does not always suffice to predictably render root canals free of bacteria. Factors such as anatomical complexities and bacterial growth as biofilm render complete disinfection of the root canal system almost impossible [4]. Therefore, in order to increase disinfection efficacy, irrigant activation systems has recently gained popularity. The current gold standard is represented by passive ultrasonic irrigation (PUI), which is effective in root canal disinfection [5]. Recently, a new mode of Er: YAG laser has been launched called Shock Wave-Enhanced Emission Photoacoustic Streaming (SWEEPS). Its efficacy is based on the delivery of pairs of ultrashort pulses (25 µs) with minimal energy levels (25 mJ) into an irrigant in the root canal [6]. The SWEEPS mode has been shown to be very promising for the removal of smear layers and debris from complex regions in the root canal [7]. However, thus far, no clinical study has evaluated the antibacterial effects of irrigant activation by SWEEPS during endodontic treatment of teeth with apical periodontitis. Thus, this study aimed to investigate the efficacy of the supplementary use of SWEEPS laser (SL) and passive ultrasonic irrigation on reducing the bacterial load and reducing inflammation detected by IL-1 β in apical periodontitis cases.

## Materials and methods

### Ethics committee approval and informed consent document

This randomized, parallel, single-blinded clinical trial was approved by the institutional ethical committee (E-10840098-772.02-3501). This study was designed according to CONSORT guidelines for reporting randomized clinical trials. This randomised clinical trial has been written according to Preferred Reporting Items for Randomized Trials in Endodontics (PRIRATE) 2020 guidelines [8]. Patients signed a printed informed consent form after explanation of the treatment procedure. Recruitment and completion of the operative procedures for the study participants were done by the principal investigator at the endodontic clinic, Faculty of Dentistry, Istanbul Medipol University in Turkey, from August 2022- February 2023.

### Patient selection and study protocol

A total of 200 patients were clinically and radiographically evaluated in terms of their conformance to the inclusion and exclusion criteria. Care was taken to ensure that the patients included in the study were over the age of 18 and medically healthy. Patients with posterior teeth diagnosed with chronic apical periodontitis and having the periapical lesions of endodontic origin with a diameter more than 3 mm, having a periapical index (PAI) score of 4 or 5, were included in the experimental phase [9]. Patients who were either pregnant, lactating, morbidly obese, presence of malignancy, presence of concomitant infection other than AP, presence of acute or chronic inflammatory disease, use of analgesics or anti-inflammatory drugs during the previous 3 months, history of trauma within 1 month that may affect IL-1beta levels, and recent use of biotin-containing vitamins were excluded from the study. In addition, patients with calcifed root canals, resorption, periodontal problems (periodontal pocket depths > 4 mm), incomplete root development, history of endodontic treatment, traumatic occlusion or excessive coronal destruction on the diagnostic radiograph taken before the procedure were not included. A total of 30 cases with negative response to electric pulp test (Parkell, NY, USA) and radiographically visible apical periodontitis lesion were equally divided into two final irrigation groups. A computer-based program (www.random.org) and simple random sampling method were used for randomization, and this procedure was performed by a researcher that was not otherwise involved in the study. Numbers were placed in dark envelopes and concealed. The envelopes were only opened when the irrigation solution was to be activated. The patients were informed about the study without specifying the group to which they were assigned. The operator discovered which activation method to use during the irrigation activation phase. The microbiologist and the biolog who performed the microbial and biochemical analyses were also blinded to the experimental groups. The clinical study was initiated after obtaining informed consent from the patients. All the root canal treatments of the patients were performed by a single endodontist with 10 years of professional experience (YEH).

The diagnosis was established according to the patient’s history, clinical inspection including palpation, tenderness to percussion, pulpal sensitivity testing, and radiographic examination. Age, gender, total number of teeth (TNT: Total number of teeth), number of root canals treated (NRC: Number of root canals), number of root canal fillings (RCF) and the presented symptoms were recorded. If the tooth is not sensitive to biting pressure but could feel different to the patient on percussion [10], it is classified as symptomatic. The number of teeth with AP and the size of the periapical lesion (PLS: Periapical lesion size) radiographically were determined by taking panoramic dental radiographs of the patients whose examination was completed (Vista Pano S-Durr Dental AG, Germany). PLS was determined by measuring the radiolucent diameter of the periapical lesion in the periapical radiographs (Carestream RVG 5200; Carestream Health Inc, Atlanta, CA, USA). PAI scoring was done using radiographic assessment of apical periodontitis [9]. In order to determine serum IL-1β levels, blood samples were taken from the antecubital vein in the sitting position after 8–10 h of fasting (08:00–09:00 in the morning) before the root canal treatment (BT) and on the third day after the treatment (AT). The blood samples taken from the patients were kept at room temperature for about 30 min and then centrifuged (10 min, 1800 g). The sera obtained after centrifugation were placed in eppendorfs and stored in the deepfreezer at − 80 °C until the working day.

### Measurement of serum IL-1β levels

On the day of analysis, serum samples stored at -80 °C were gradually thawed by keeping them in the refrigerator at + 4 °C for 12 h. All samples were kept at room temperature for 30 min before the study. BT lab branded Human Interleukin-1beta (IL-1β) ELISA Kit (Shangai, China) was used to measure serum IL-1β levels before and after treatment in patients who underwent SL or PUI. The kit has a sensitivity of 10.07 pg/mL with a measuring range of 20-6000pg/mL and an within-run coefficient of variation (CV) < 5%. IL-1β levels were measured in an ELISA device (BioTek Epoch 2 Microplate ELISA Reader, USA).

### Root canal treatment and microbiological sampling procedures

After applying local anesthesia (Articaine 4% with 1:200,000 epinephrine, Ultracaine DS Fort, Hoechst-Marion Roussel, Frankfurt, Germany), the tooth was isolated with a rubber dam. The rubber dam retainer, and the area of the rubber dam surrounding the tooth was disinfected by swabbing with 30% hydrogen peroxide and then 2.5% NaOCl solution, followed by inactivation with 10% sodium thiosulfate in order to avoid interference with bacteriological sampling. The sterility of the external surfaces of the crown was checked by taking a sample from the crown surface and this was the sterility control total bacteria sample (SC). Paper points were transferred to cryotubes containing phosphate-buffered saline (PBS) solution stored at − 20 °C. In each case, a single root canal was sampled in order to confine the microbial evaluation to a single ecological environment. In multirooted teeth, the root with the periapical lesion was selected. If there were periapical lesions in all roots, the wider canal was selected.

At the beginning of the AP treatment, a sample was taken from the canal of the tooth to determine the number and type of bacteria in the environment. This sample was designated S1-. A second sample, defined as S2, was taken after standard root canal treatment. In addition to standard treatment, a third sample was taken after SL or PUI was performed (S3).

Access cavities were opened using sterile diamond and carbide burs. Working length was determined using an electronic apex locator (Propex Pixi, Denstply Sirona, Ballagues, Switzerland) and then periapical radiographs were taken to ensure that a size #10 K-type file has reached the radiographic apex. Irrigation with sterile saline solution was performed in order to moisten the canal prior to sample collection. Next, the canal was left filled with saline, and a small hand instrument was placed at the WL and used to gently file the canal walls. An initial microbiologic sample (S1) was taken from the root canal with sterile paper points consecutively placed at the WL. Three sterile paper points were placed individually inside the root canal for 1 min each to collect the initial content of bacteria. Then the paper points and the endodontic hand instrument, without the handle, were transferred to cryotubes containing 300 µl of PBS solution stored at − 20 °C. The samples were transferred to genetic analysis laboratory for further analysis in cold chain.

Root canals were prepared by using the ProTaper Next files (Dentsply-Sirona, Ballaigues, Switzerland) and irrigated with 2.5% NaOCl (300 rpm, 200 gcm and with brushing motion). The canals were apically enlarged to size 40 (X4) at the working length (SX, X1, X2, X3, and X4, respectively). Between each instrument change, the root canal was irrigated with 5 ml of 2.5% NaOCl solution. Hence, a total of 25 ml of the irrigating solution was used. After instrumentation was completed, the smear layer was removed with 2 ml 17% ethylenediamine tetra-acetic acid (EDTA), which was left in the canal for 3 min, followed by 2.5% NaOCl. Then, saline solution was used to flash the root canals to eliminate the effect of EDTA. The root canal was dried with sterile paper points and flushed with 2 ml of 10% sodium thiosulfate for 1 min to inactivate the NaOCl solution. Next, a sample (S2) was taken from the canals as described for S1.

After taking of S2 sample, 3 ml of 2.5% NaOCl and 2 ml of 17% EDTA for the final irrigation process were carried out for 1 min with each irrigant. Saline solution was activated in the root canal for 20 s between two solutions and used to flash the root canals to eliminate the effect of EDTA in all groups. SWEEPS group: The SWEEPS fiber tip (25 µs ultra-short dual pulse model Auto SWEEPS mode) was inserted into the Er: YAG laser source (2,940 nm, 20 mJ per pulse, 15 Hz, 0.3 W power, and 50 µs pulse frequency). The SWEEPS tip placed in the access cavity was kept in a stable position, and activation was performed in the SWEEPS mode. 3 ml of 2.5% NaOCl solution was activated in three periods of 20 s (1 ml of 2.5% NaOCl in each cycle). Then, the same procedure was repeated with 2 ml of 17% EDTA solution.

PUI group: The irrigation solution was used by adapting an ultrasonic tip (IRRI S 21/25; VDW, Munich, Germany) to an ultrasonic device. The power setting of the ultrasonic device was 30% (VDW Ultra; VDW, Munich, Germany). The tip was activated a total of three times, with each cycle lasting 20 s and involving the use of 1 ml of 2.5% NaOCl. Then, 2 ml of 17% EDTA solution was activated for 1 min as describe above. The ultrasonic tip was placed 2 mm behind the working length without the ultrasonic file binding to the canals walls.

In both groups, 2.5% NaOCl was inactivated again using 10% sodium thiosulphate, and a new sample was taken after the final irrigation protocol (S3). After S3 samples were taken, the root canals were dried using paper points and filled with the root filling using lateral condensation of gutta-percha and AH Plus sealer (Dentsply DeTrey). The access cavities were restored with composite resin, and a final radiograph was taken.

### Genomic DNA isolation and measurement of DNA concentration

DNA was extracted using the QIAamp DNA Mini Kit (Qiagen, Germany) following the protocol recommended by the manufacturer [11]. Before DNA isolation, samples (the tubes with paper points) were digested at 50–60 °C by vortexing for 30s every 10 min in order to ensure disaggregation of all bacteria into the PBS solution. Afterwards, the paper points were aseptically removed from the suspension and the bacterial suspension was pelleted by centrifugation for 10 min at 5000 g. The pellet was then resuspended in 180 µl buffer ATL supplied by QIAamp DNA Mini Kit (QIAGEN GmbH, Hilden, Germany) and 20 µl proteinase K (20 mg/ml) was added. Samples were incubated for 3 h at 56 °C. Subsequently, total bacterial genomic DNA was isolated according to the protocol of the QIAamp DNA Mini Kit. The final volume of DNA solution of each sample was 150 µl and was taken into account during calculation. DNA concentration (absorbance at 260 nm) was determined with a spectrophotometer (Promega Quantifluor).

### Amplification of 16 S rRNA genes

Primers for Universal and *Streptococcus* 16 S rRNA genes were designed in this study. After DNA extraction of samples with QIAamp DNA Mini Kit, 700–800 bp of 16 S rRNA sequences were amplified by using universal E8F forward primer (5′-AGAGTTTGATCCTGGCTCAG-3′) and universal E1115R reverse primer (5′-AGGGTTGCGCTCGTTG-3′). The final volume of PCR reactions for each isolated bacterial strain was adjusted to 25 µl. The amplification reactions of 16 S rRNA genes were performed with the following conditions. 1 cycle of predenaturation at 95 °C for 3 min, 35 cycles of 95 °C for 30 s, 55 °C for 30 s and 72 °C for 60 s which continue with a final extension step at 72 °C for 10 min. The PCR products were analysed by electrophoresis using 2% agarose gel (containing ethidium bromide) in Tris/BoratE/EDTA (TBE) buffer, with gels being analysed under ultraviolet light (at 140 V for 20 min). Their images were visualised under ultraviolet illumination. In addition, the control and optimisation of primers to be used for droplet digital PCR (ddPCR) was also done in conventional PCR.

### Purification and sequencing of the 16 S rRNA gene

After the PCR reactions, the purification of PCR products is done by hydrolysing the excess primers and nucleotides with ExoSap-IT (Thermo, PN: 78201.1.ML) containing Exonuclease I and Alkaline Phosphatase enzymes. 2 µl of ExoSap-IT was mixed with 5 µl of PCR product for each sample. The ExoSap reaction is performed at 37 °C for 15 min (enzyme activation) followed by 15 min (inactivation) at 80 °C. Sequencing reactions were performed by using Bigdye™ Terminator v3.1 cycle sequencing kit (Thermo). The reactions were performed according to the kit manual for all isolated strains. After purification of the products with Exosap, the sequence reaction was performed with BigDye Terminator v3.1 Cycle Sequencing Kit (Thermo) under the following conditions. After the sequence PCR, BigDye products were purified by colon method. Zymo ZR DNA Sequencing Clean-up Kit (Zymo Research, USA) was used for this process. All samples were purified in accordance with the protocol given in the kit and executed on the 3130XL genetic analyzer.

### Droplet digital PCR

Droplet Digital PCR (ddPCR) was performed using primers designed according to the 16S rRNA region specific to the total bacteria and *Streptococcus* species, after sequencing, absolute quantitation from the bacterial species found in the paper-point sample. Primer pairs were 16S-F-5’-AGGGAATCTTCSGCAATGGG-3’) and 16 S-R-5’-ACGCCCAATAAATCCGGACA-‘3 for total bacteria and Strep-F-5’- GAGTACGACCGCAAGGTTGA − 3’ and Strep-R-5’- ACCTGTCTCCGATGTACCGA − 3’ primer pairs for *Streptococcus* species. For absolute quantitation of *Streptococcus* and total 16 S rRNA, PCR was performed with two primer pairs from the same sample. 22 µl of PCR mix containing 8 µl of EvaGreen mix (Bio-Rad, cat.no. 1,864,034), 11.5 µl of dH2O, 0.5 µl of both forward and reverse primer and 1.5 µl of DNA from each sample. Thermal cycling conditions were: 95 °C for 5 min, then 35 cycles of 95 °C for 30 s and 60 °C for 1 min and two final steps at 4 °C for 5 min and 90 °C for 5 min with a 4^◦^C infinite hold. After PCR was completed, the sealed plate was transferred into the plate holder of the QX200 Droplet Reader (Bio-Rad, cat. no. 1,864,003).

### Statistical analysis

SPSS statistics version 25 (IBM, USA) and MedCalc^®^ software (version 15.8, Belgium) were used for statistical analysis of all data of the study. Chi-square Test was used to compare the categorical data of two independent groups in the study. The Unpaired *t* test was used to compare the parametric data of two independent groups, while the Mann-Whitney *U* Test was used to compare the nonparametric data. Pearson correlation analysis in parametric data and Spearman correlation analysis in nonparametric data were used to investigate the relationship between serum pretreatment IL-1β, PAI score, RCF, total number of teeth (TNT: Total number of teeth) and PLS. Bar plot graphics were used to display nonparametric data and box plot graphics were used to display parametric data.

### Power analysis and sample size calculations

Priory power analysis (PS Power and sample Size Program, Version 3.1.2) was performed with the data of a recent study [12] comparing IL-1β levels in fluid taken from healthy and periodontal diseased areas. The minimum sample size was calculated for this study by taking α: 0.05 and power: 0.80. It was determined that in order to reject the null hypothesis, it was necessary to work on 4 experimental and 4 control subjects for the study, which would consist of two groups. Experimental group 15 and control group 15 were determined in this study to ensure sufficient statistical power and robustness of the results.

## Results

The patients who underwent SL and PUI in addition to standard treatment for AP were not statistically different in terms of age (32 ± 11 and 33 ± 11 years, respectively) and gender (M/F: 7/8 and 8/7, respectively) (*p* > 0.05) (Table 1). There was no statistical difference between the SL and PUI groups with AP in terms of the number of symptomatic cases (40% and 33%, respectively, *p* > 0.05). Again, there was no difference between SL and PUI groups in terms of RCF, number of fillings (NF), missing teeth and PAI scores (*p* > 0.05).

**Table 1:** Demographic, medical history and clinical examination of patients who underwent SWEEPS laser or passive ultrasonic irrigation for root canal treatment

	SL Group	PUI Group	*p* value
n	15	15	-
Gender, F (%)	8(53%)	7(47%)	^a^ 0.715
Age, year	32 ± 11 29(18–58)	33 ± 11 31(18–54)	^b^ 0.901
AP with Symptoms, n (%)	6(40%)	5(33%)	^a^ 0.705
RCF, n	2 ± 2 1(0–7)	2 ± 2 1(0–7)	^b^ 0.479
NF, n	4 ± 3 3(0–11)	4 ± 3 4(1–12)	^b^ 0.884
MCTN-AP, mode (f)	36(3), 46(2), 47(2)	34 (3), 46(2), 36(2)	^a^ 0.628
Missing teeth, n	2 ± 3 0(0–13)	2 ± 3 1(0–9)	^b^ 0.544
PAI, score	4 ± 1 4(4–5)	4 ± 1 4(4–5)	^b^ 0.767

a Chi-square Test, b Mann-Whitney Test

In statistical comparisons, non-parametric data were shown as mean ± standard deviation and median (min-max). F: Female, AP: Apical periodontitis, SL: SWEEPS laser, PUI: Passive ultrasonic irrigation, RCF: Number of root canal filling, NF: Number of fillings, MCTN-AP: The most common tooth number with AP, mode: Most common number in a dataset, f: Frequency, PAI: Periapical index

It was determined that the tooth with the most common AP (MCTN-AP) in the SL group was tooth 36 (mode) and its frequency was 3 in the group of 15 people (Table 1). Again, the incidence of AP was found to be 2 in teeth 46 and 47. In the PUI group, MCTN-AP was found to be tooth 34 (mode) and the frequency of AP incidence of tooth 46 and 36 was 2. There was no statistical difference between the two study groups in terms of teeth with the most common AP (*p* > 0.05).

**Fig. 1:**
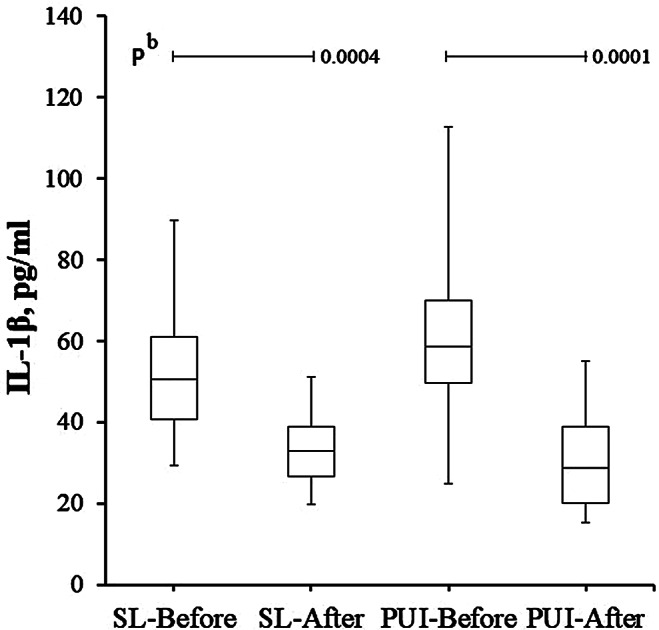
Nonparametric bar plot graph showing IL-1β levels before and three days after SL and PUI applications. It is observed that there is a statistically significant decrease in IL-1β levels after SL and PUI administration. ^c^ Mann-Whitney Test, SL: SWEEPS laser, PUI: Passive ultrasonic irrigation

The difference between PLS values of randomly selected SL and PUI groups was statistically insignificant (*p* > 0.05) (Table 2). Again, there was no difference between the groups in terms of IL-1β levels before and after treatment (*p* > 0.05). However, pre-treatment IL-1β levels were statistically higher than posttreatment levels in both the SL group and the PUI group (*p* < 0.001) (Fig. 1).

**Table 2:** Radiographic and biochemistry tests of patients who underwent SWEEPS laser or passive ultrasonic irrigation for root canal treatment

	SL Group	PUI Group	*p* value
N	15	15	-
PLS, mm	11 ± 4 10(4–18)	10 ± 4 11(4–17)	^b^ 0.493
IL-1β-BT, pg/ml	53 ± 18 51(29–90)	60.7 ± 21.6 58.5(24.8-112.7)	^b^ 0.345
IL-1β-AT, pg/ml	33.1 ± 9.1 32.8(19.9–51.2)	31.0 ± 13.0 28.6(15.3–55.0)	^b^ 0.494
*p value	^b^ 0.0004	^b^ 0.0001	

b Mann-Whitney Test, * p value between before and after-treatment IL-1β level of the group

In statistical comparisons, non-parametric data were shown as mean ± standard deviation and median (min-max). SL: SWEEPS laser, PUI: Passive ultrasonic irrigation, AP: Apical periodontitis, PLS: Periapical lesion size, IL-1β-BT: Before treatment interleukin-1beta, IL-1β-AT: After treatment interleukin-1beta

**Fig. 2:**
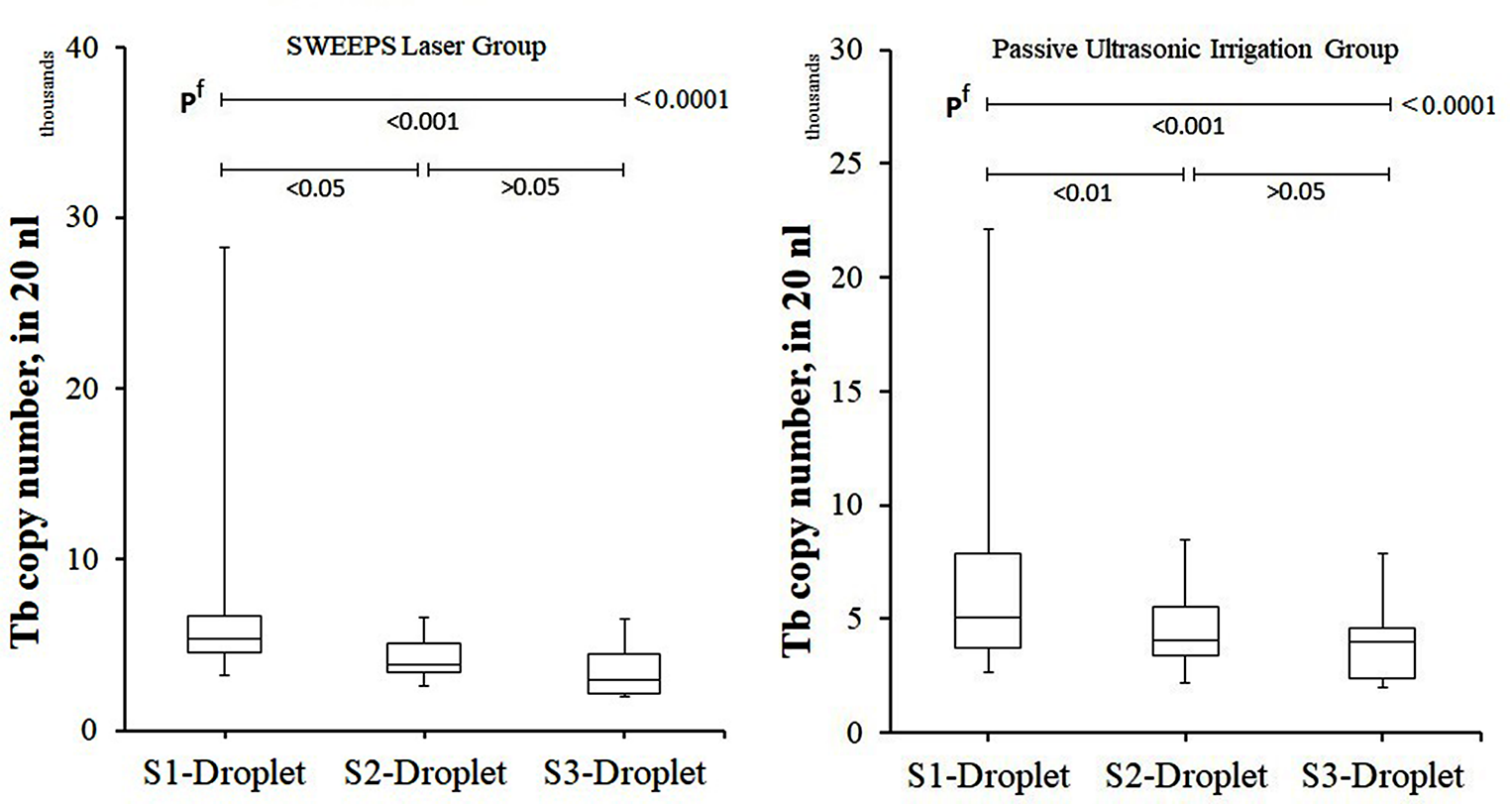
Bar plot graph of the total bacterial copy numbers detected in the samples taken at S1, S2 and S3 stages in patients with AP who underwent SL and PUI. It is observed that there is a statistically significant difference between S1 and S2 and S3 in terms of total bacterial count. There is no significant difference between S2 and S3. f Friedman Test (Nonparametric Repeated Measures ANOVA), SL: SWEEPS laser, PUI: Passive ultrasonic irrigation, Tb: Total bacteria

**Fig. 3:**
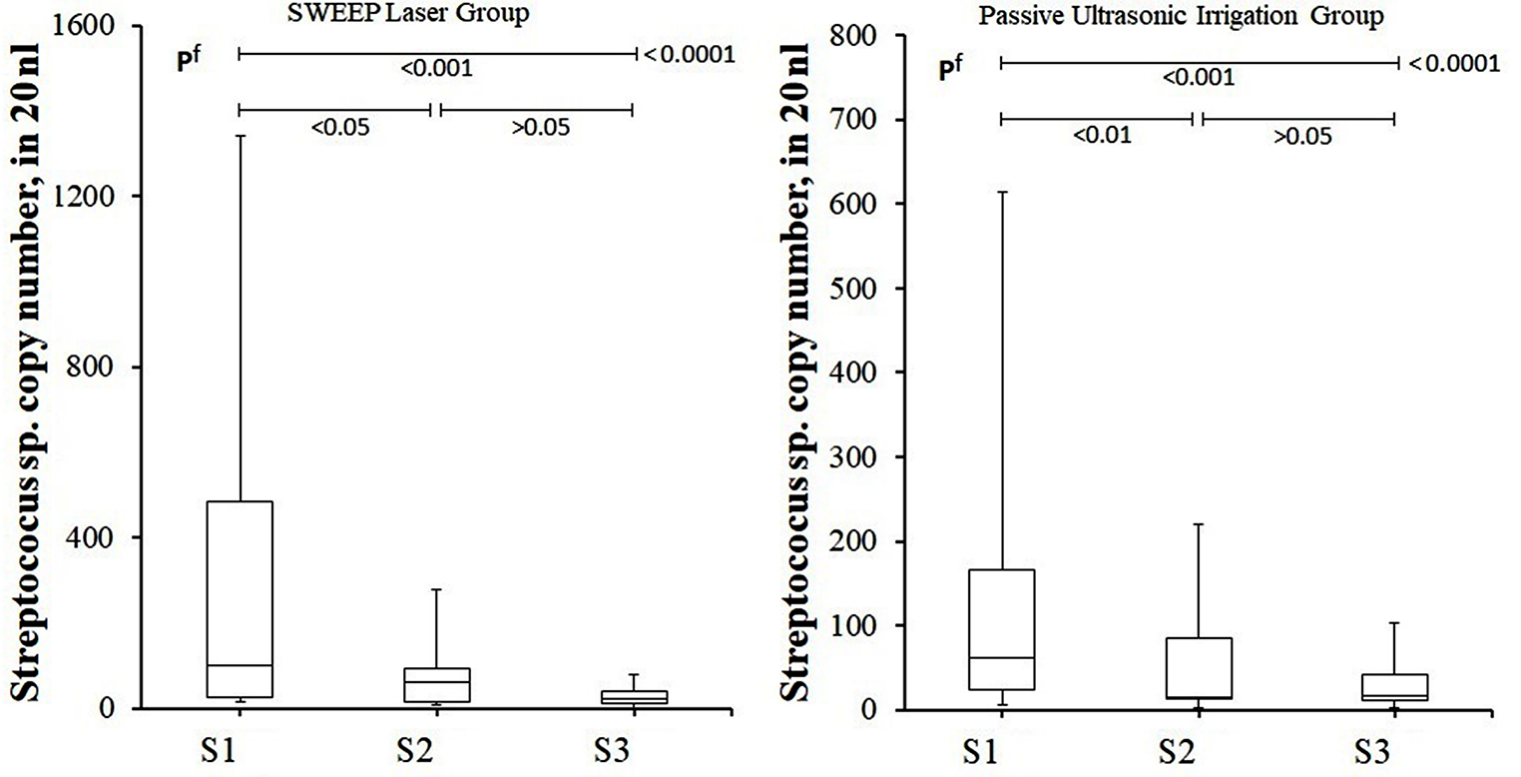
Bar plot graph of *Streptococcus* species copy numbers detected in samples taken at S1, S2 and S3 stages in patients with AP who underwent SL and PUI. It is observed that there is a statistically significant difference between S1 and S2 and S3 in terms of the number of *Streptococcus* species. There is no significant difference between S2 and S3. f Friedman Test (Nonparametric Repeated Measures ANOVA)

In this study, all sterility controls yielded negative results [sterility control total bacteria (SC-Tb) and sterility control *Streptococcus* copy number (SC-St)]. No statistical difference was found between SL and PUI groups in terms of SC-Tb, S1-Droplet, SC-St and S1-St copy numbers (*p* > 0.05) (Table 3). In addition, there was no difference between the groups in terms of copy numbers of S2-Droplet, S3-Droplet, S2-St, S3-St (*p* > 0.05). Contrary to these results, total bacterial copy number and *Streptococcus* sp. bacterial copy numbers decreased gradually in the S1, S2 and S3 stages in both treatment groups and the decrease was statistically significant (*p* < 0.001) (Figs. 2 and 3). When the SL and PUI groups were compared in terms of the decrease in the total bacterial copy number by considering the difference between the S2 and S3 stages, it was determined that there was no difference between the two applications [Mann-Whitney *U* test, median (min-max): 19% (12 – 43%) vs. 15% [(-12%) – 41%], *p* = 0.590, respectively].

**Table 3:** Total bacteria copy number of patients who underwent SWEEPS laser or passive ultrasonic irrigation for root canal treatment

	SL Group	PUI Group	*p* value
N	15	15	-
SC-Tb, nl	14 ± 4 14(8–22)	13 ± 4 12(9–22)	^b^ 0.724
S1-Droplet number, nl	6973 ± 6050 5400(3240–28,200)	6931 ± 5148 5080(2640–22,140)	^b^ 0.756
S2-Droplet number, nl	4247 ± 1149 3800(2560–6600)	4602 ± 1774 4040(2200–8500)	^b^ 0.740
S3-Droplet number, nl	3407 ± 1416 2960(2000–6500)	3806 ± 1669 4020(2000–7880)	^b^ 0.507
SC-St copy number, nl	0.6 ± 0.6 0.4(0.0–2.0	0.5 ± 0.4 0.5(0.0-1.6)	^b^ 0.983
S1-St copy number, nl	325 ± 403 100(14-1342)	151 ± 203 62(6-614)	^b^ 0.237
S2-St copy number, nl	70 ± 71 60(10–278)	53 ± 59 15(2-220)	^b^ 0.319
S3-St copy number, nl	26 ± 21 22(0–78)	28 ± 26 16(2-102)	^b^ 0.983

b Mann-Whitney Test, * p value between before and after-treatment IL-1β level of the group

In statistical comparisons, non-parametric data were shown as mean ± standard deviation and median (min-max). SL: SWEEPS laser, PUI: Passive ultrasonic irrigation, AP: Apical periodontitis, IL-1β-BT: Before treatment interleukin-1beta, IL-1β-AT: After treatment interleukin-1beta, PLS: Periapical lesion size, SC-Tb: Sterility control total bacteria copy number, S1-Droplet number: S1-Total bacteria copy number (in 20 nanoliter: nl), S2-Droplet number: S2-Total bacteria copy number (in 20 nanoliter: nl), S3-Droplet number: S3-Total bacteria copy number (in 20 nanoliter: nl), SC-St: Sterility control streptococcus species, S1-St: S1-Streptococcus species, S2-St: S2-Streptococcus species, S3-St: S3-Streptococcus species

Table 4 shows the most similar bacterial species based on the BLAST analysis results. In this study, the main bacterial species detected in the samples taken at S1 stage in both SL and PUI groups was mostly *Streptococcus* species (Table 4). *Lactobacillus* and *Enterococcus* sp were determined as the main bacterial species at S2 and S3 stages, respectively.

**Table 4:** Prevalence of main bacterial species in S1, S2 and S3 samples

	SP Group			PUI Group		
No	S1	S2	S3	S1	S2	S3
1	Streptobacillus sp-89%	Lactobacillus sp-95%	Lactobacillus sp-93%	Enterococcus sp-89%	Lactobacillus sp-87%	Enterobacter sp-91%
2	Pseudomonas sp-77%	Lactobacillus sp-89%	Sphingobac. sp- 85%	Streptococcus sp-90%	Streptococcus sp-95%	Enterococcus sp-89%
3	Streptococcus sp-94%	Streptococcus sp-89%	Enterobac. sp-92%	Streptococus sp-88%	Lactobacillus sp-91%	Streptococcus sp-83%
4	Streptococcus sp-96%	Lactobacillus sp-96%	Lactobacillus sp-95%	Fusobacterium sp-88%	Enterococcus sp-98%	Enterococcus sp-97%
5	Streptococcus sp-89%	Enterococcus sp-90%	Enterococcus sp-87%	Enterococcus sp- 94%	Flavobacterium sp-88%	Fusobacterium sp-89%
6	Enterobacter sp-92%	Lactobacillus sp-96%	Enterococcus sp-93%	Streptococcus sp-92%	Streptococcus sp-89%	Streptococcus sp-92%
7	Streptococcus sp-91%	Enterococcus sp-93%	Pseudomonas sp-95%	Enterobacter sp-93%	Enterobacter sp-83%	Fusobacterium sp-92%
8	Lactobacillus sp-93%	Enterococcus sp-90%	Lactobacillus sp-94%	Streptococcus sp-93%	Enterococcus sp-89%	Streptococcus sp-86%
9	Streptococcus sp-94%	Lactobacillus sp-9%6	Streptococcus sp-90%	Lactobacillus sp-93%	Enterobacter sp-88%	Streptococcus sp-86%
10	Fusobacterium sp-95%	Enterococcus sp-93%	Lactobacillus sp-94%	Lactobacillus sp-90%	Lactobacillus sp-95%	Enterococcus sp-94%
11	Streptococcus sp-87%	Lactobacillus sp-%89	Sphingobac. sp. 85%	Enterococcus sp-94%	Lactobacillus sp-88%	Fusobacterium sp-92%
12	Lactobacillus sp-91%	Lactobacillus sp-%89	Streptococcus sp-85%	Lactobacillus sp-93%	Sphingobac. sp-88%	Enterococcus sp-87%
13	Streptococcus sp-68%	Enterococcus sp-%88	Sphingobac. sp -94%	Enterococcus sp-92%	Streptococcus sp-90%	Enterococcus sp-89%
14	Streptococcus sp-93%	Streptococcus sp-%84	Streptococcus sp-86%	Lactobacillus sp-90%	Sphingobac. sp-90%	Enterococcus sp-93%
15	Enterococcus sp-98%	Fusobacterium sp-%98	Enterococcus sp-93%	Streptococcus sp-75%	Lactobacillus sp-91%	Enterococcus sp-85%

SP: SWEEPS laser, PUI: Passive ultrasonic irrigation, Enterobac.: Enterobacteriaceae, Sphingobac.: Sphingobacterium, sp: Species

**Fig. 4:**
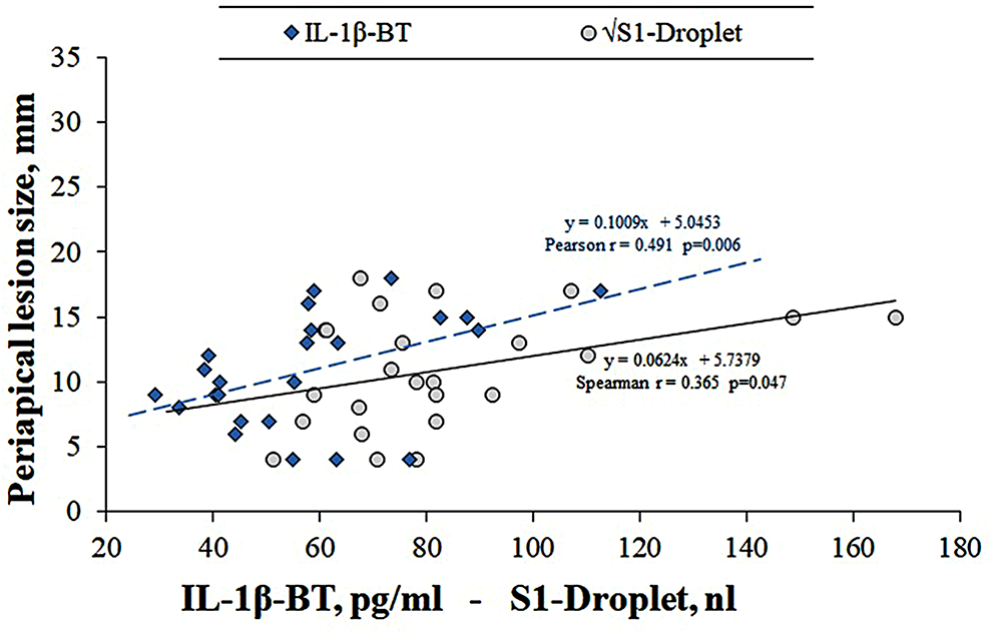
Scatter plot showing the relationship between periapical lesion size and pretreatment IL-1β levels and S1-Droplet copy number. It is seen that there is a moderate positive correlation between the variables

According to the correlation matrix results (Table 5), a positive correlation was observed between PLS and IL-1β-before treatment (BT) and S1-Droplet (*r* = 0.491 and *r* = 0.379, respectively). When these results are reanalyzed with appropriate statistical methods; the correlation between PLS and IL-1β-BT and S1-Droplet values was found to be statistically significant (Pearson *r* = 0.7414, 95% CI: 0.52 to 0.869, *p* < 0.001 and Spearman *r* = 0.365, 95% CI: -0.006, respectively). to 0.647, *p* = 0.047) (Fig. 4).

**Table 5:** Correlation Matrix outputs using Pearson correlation of the independent variables of the study

	A:	B:	C:	D:	E:	F:	G:	H:	I:	J:
**A: PLS**	1.0000									
**B: IL-1β-BT**	**0.4906**	1.0000								
**C: SC-Tb**	-0.0155	-0.0117	1.0000							
**D: S1-Droplet**	**0.3795**	**0.3548**	0.0351	1.0000						
**E: S2-Droplet**	0.2726	0.1982	-0.0165	**0.6959**	1.0000					
**F: S3-Droplet**	0.2983	0.2644	0.0388	**0.7785**	**0.8923**	1.0000				
**G: SC-St**	-0.1809	0.1787	0.0900	0.2472	0.0017	0.0235	1.0000			
**H: S1-St**	0.2856	0.2529	0.0759	0.0660	0.0133	-0.0051	0.0241	1.0000		
**I: S2-St**	0.0662	0.2281	0.1459	-0.0034	-0.1087	-0.0609	0.2370	**0.6039**	1.0000	
**J: S3-St**	-0.1260	0.1696	-0.0577	-0.2553	-0.2727	-0.2707	-0.2992	**0.5077**	**0.3887**	1.0000

Significant correlations (> 0.30) are reanalyzed according to the nature of the data (parametric or nonparametric) using appropriate statistical methods. Correlation values are characterized as; 0.30–0.50 low, 0.50–0.70 moderate, > 0.70 high correlation (Hinkle et al. 2003). IL-1β-BT: Before treatment interleukin-1beta, PLS: Periapical lesion size, SC-Tb: Sterility control total bacteria copy number, S1-Droplet: S1-Total bacteria copy number (in 20 nanoliter: nl), S2-Droplet: S2-Total bacteria copy number (in 20 nanoliter: nl), S3-Droplet: S3-Total bacteria copy number (in 20 nanoliter: nl), SC-St: Sterility control streptococcus species, S1-St: S1-Streptococcus species, S2-St: S2-Streptococcus species, S3-St: S3-Streptococcus species

Similarly, correlations between S1-Droplet and S2-Droplet and S3-Droplet in Table 5 were found to be highly positive (Spearman *r* = 0.814, 95% CI: 0.635 to 0.910, *p* < 0.001 and Spearman *r* = 0.7274, 95% CI: 0.489 to 0.865, *p* < 0.001, respectively). Again, good positive correlations between S1-St and S2-St and S3-St copy numbers were also statistically significant (Spearman *r* = 0.7923, 95% CI: 0.598 to 0.899, *p* < 0.001 and Spearman *r* = 0.676, 95% CI: 0.407 to 0.837, *p* < 0.001) [13].

## Discussion

Apical periodontitis is a disease characterized by tissue liquefaction and pulpal devital lesions resulting from infection and inflammation of the periapical alveolar bone of the tooth with microbial organisms. Advanced stages of AP are characterized by a radiolucent image in the apical zone and enlargement of the periodontal membrane on radiographs as a result of deterioration of the tooth’s cortical bone. In the etiopathology of AP formation; oral microbiota and host defense system cells, effectors and antibodies play the main role [14–18]. Therefore, studies that will provide a good understanding and characterization of the microbial environment causing AP will increase the success of AP treatment. In this study conducted for these purposes, types of bacteria in the microbial environment and their amount as well as IL-1β, which is an important trigger of the inflammatory process, were observed at different stages of AP treatment. Moreover, the evaluation of the efficacy of adjunctive approaches by both SL and PUI applications in addition to AP treatment with standard chemomechanical preparation made the study unique.

In general, one of the biggest mistakes made when comparing treatment methods is that the demographic and clinical characteristics of the study groups are not selected similarly. Therefore, these factors affecting the results of the study may cause erroneous interpretation of the research outputs [19, 20]. In this study, it was found that the groups were similar in terms of age, gender, symptomatic teeth, RCF, NF, missing teeth and PAI scores (or at least there was no difference between the groups in terms of these characteristics), being able to determine which of the adjunctive approaches (either SL or PUI) in addition to the chemomechanical preparation was more effective was an important advantage of this study. The fact that both groups were similar in terms of PLS, along with the most common AP observed tooth number, which is also an indicator of the localization of AP, made the traceability of the effects due to SL or PUI application more reliable.

The physiopathological process of AP is determined by the encounter between microbial organisms and the host defense system [15, 18, 21]. The host response includes a variety of cells (polymorphonuclear leukocytes, lymphocytes, monocytes/macrophages, and plasma cells), mediators, effector molecules, and antibodies. However, since polymorphonuclear leukocytes also secrete cytoplastic granules (lytic enzymes), they can cause structural damage to tissue cells and extracellular matrix [14]. Within the defense mechanism, the nature of the tissue forming the structure (collagen, fibroblasts, odontoblasts and mesenchymal cells) as well as localization and environmental factors are determinative in the formation of the lesion [14]. Behavioral aspects and hygienic measures play a big role in oral and dental health [22]. Apical periodontitis, which can occur in different positions, may be caused by infectious or aseptic inflammation [15, 22]. The fact that the most common tooth with AP in this study was number 36 and 34 according to the groups, and the second most common tooth with AP was tooth number 46, which is in line with the above literature findings.

Proinflammatory cytokines such as IL-1β and tumor necrosis factor (TNF)-α, which are strong stimulators of inflammation and tissue damage, play important roles in AP. Especially, IL-1β promotes the production of proteinases that accelerate bone resorption. In studies conducted for this purpose, significant increases in blood IL-1β levels were observed in most patients with chronic periodontitis [23–25]. It has been reported that AP may cause an increase in IL-1β both locally (in gingival fluid and saliva) and systemically (in the blood). In addition, some researchers reported that susceptibility to periodontitis may increase or disease progression which is further accelerated in patients with IL-1β polymorphism [26, 27]. IL-1β, which triggers a series of inflammatory reactions and bone resorption, has become a therapeutic target for autoimmune and autoinflammatory diseases such as rheumatoid arthritis, gout and type II diabetes mellitus in addition to periodontitis [28]. In this study, which we conducted in the light of the above information, IL-1β levels were compared between the groups and before and after treatment in order to test the treatment effectiveness of SL or PUI application. While there was no difference between the groups, the decrease in posttreatment IL-1β levels in both SL and PUI groups compared to pre- treatment levels was evaluated as evidence that these two applications have similar efficacy. Moreover, the reporting that there is no difference between the results of SL and PUI application in many recent studies also supports our results [29–32]. Therefore, all these findings show that IL-1β plays an important role in the pathophysiology of AP. In addition, these results showed that the efficacy of AP treatment can be followed by IL-1β levels.

The direct proportional relationship between periapical lesion size and the number of S1-total bacteria and IL-1β-BT levels in this study shows that the proinflammatory process is determinant in the pathophysiology of the lesion in AP. In addition, it is understood that amount of bacteria has an important contribution to this formation.

**Fig. 5:**
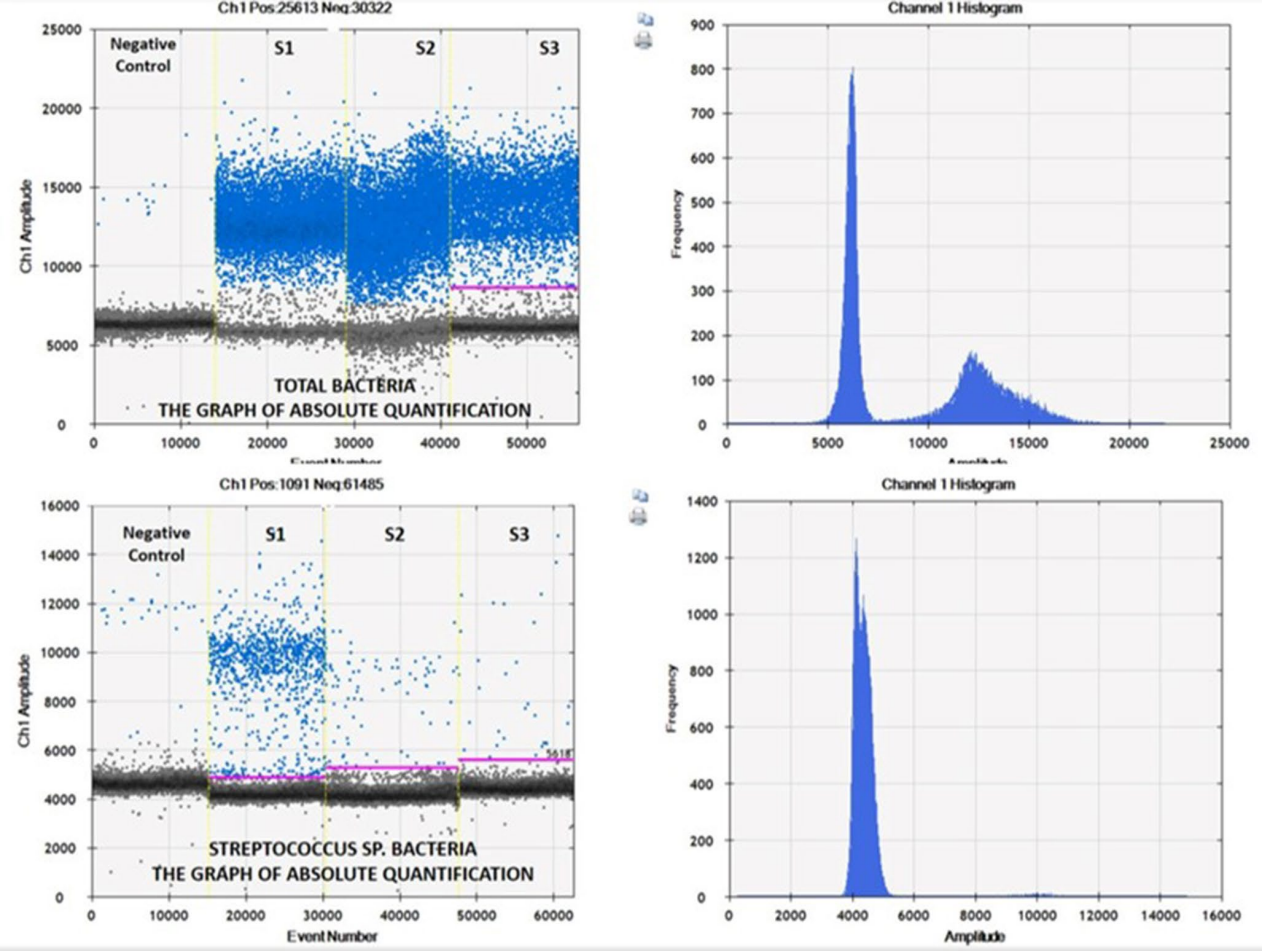
Absolute quantification graphics for both total bacteria and *Streptococcus* species

There are hundreds of species of microorganisms in the microbiota of the oral cavity. Therefore, the absence of important commensal bacteria necessary for a healthy symbiotic relationship that prevents opportunistic pathogen overgrowth or the presence of a population of pathogenic bacteria are often precursors of the occurrence of periapical disease [33, 34]. Few microorganisms in the dental plaque and their metabolic products infiltrate the apical periodontium through the root canals and the pathological gingival pocket [35]. In this way, microorganisms can lead to inflammation and subsequent complications such as pulpitis and acute or chronic inflammation of the periapical tissues. Treatment of the developing AP lesion includes chemo-mechanical preparation of the canals, use of antibacterial medicaments, and hermetic root canal filling [21]. Therefore, having knowledge about the microbial species of different endodontic infections will increase the effectiveness of the treatment. This increases the diagnostic value of microbial tests with root canal samples for microbiological diagnosis [36, 37]. In the light of this information, in this study, microbial diversity was determined by taking samples from the root canal of the tooth with AP at various stages (S1, S2 and S3) and sequencing of 16 S rRNA genes was done using Sanger sequencing method. PCR reaction was performed with 16 S rRNA gene-specific primer pairs. Agarose gel electrophoresis was performed and the result of the reaction was examined to see if the target sites were successfully amplified. The most common bacterial species in microbiota were determined by the BLAST analysis. The bacteria that cause the most common infections in endodontics are listed as *Streptococcus* species and *Enterococcus faecalis* in the literature [38–40]. In direct connection with the literature, it can be said that two bacterial species were encountered in our research. In this study, total bacteria and *Streptococcus* sp. quantity has been determined. After the quantification experiments, the results were compared with the results of the sequence analysis. Quantification and sequencing results were in agreement with each other in almost all samples (Fig. 5). There were similarities between SL and PUI groups in terms of SC-Tb, S1-Droplet, SC-St and S1-St copy numbers and the main bacterial species (*Streptococcus* sp) was the same in both groups at the S1 stage. It shows that a favorable environment is provided for researching and finding which approach (SL or PUI) is more beneficial.

In both groups, chemo-mechanical procedures using rotary instruments and 2.5% NaOCl irrigation resulted in a significant decrease in bacterial copy number compared to pretreatment (S1). The drastic reduction in the amount of bacteria from S1 to S2 samples can be explained by the mechanical action of the rotary instrument along with the chemical properties and the flow of the 2.5% NaOCl solution. This data is in accordance with previous clinical studies evaluating the effectiveness of chemo-mechanical procedures using 2.5% NaOCl irrigation in reducing the number of bacteria [41–43]. Intragroup comparisons showed that irrigation activation promoted a substantial reduction in bacterial load in root canals. This agrees with other studies that showed that irrigant activation promote higher bacterial elimination [44, 45]. This may be a result of several factors. Activation increases the chances for the solution to touch more canal wall surfaces and a larger volume of irrigants reaching the apical segment, and better irrigant exchange in this region^44^ and thus is more effective in removing adhering biofilms and infected dentin. Moreover, the activation results in a higher probability of solution incorporating anatomic irregularities, fins, and recesses. In addition, the fact that there was no difference in the copy number of S2-Droplet, S3-Droplet, S2-St, S3-St shows that SL and PUI applications are not superior to each other in terms of reducing the bacterial load. In this study, we calculated the amount of *Streptococcus* sp. reduction for S1, S2 and S3, and as expected, this pathogenic organism decreased in both groups. There were no significant differences between the 2 irrigation groups in reducing the streptococcal counts. *Streptococcus* species in S1 samples were either completely or very strongly reduced after irrigation in S2 and activation in S3. Again, the finding that the copy numbers of total bacteria and *Streptococcus* species decreased at similar rates in the S1, S2 and S3 stages in both treatment groups indicates that the standard treatment approach and additional SL and PUI applications were successful. As a result, these findings showed that both activation methods had similar efficacy in reducing bacterial load.

In this study, posterior teeth including both molars and premolars were preferred due to the complex structure of the pulp anatomy and difficulty of accessing extra root canals [46]. There are studies in the literature that draw attention to the high failure rates of root canal treatment in posterior teeth [47]. 16 of the included teeth were premolars, 12 lower premolars and 4 upper premolars. While 14 of the teeth were molars (12 lower molars and 2 upper molars). All teeth had straight root canal anatomy (< 20°) and chronic apical periodontitis lesions with a diameter more than 3 mm, having a periapical index (PAI) score of 4 or 5. Breakdown of apical constriction is observed in nearly all of the cases. Initial apical file was #25 in 22 of the cases, while it was #20 in remaining cases. Our aim was to prepare apical size at least three file sizes larger than the first file that bound at the apex. As varied apical size could affect the penetration of irrigants in the apical third, debridement and disinfection [48], the apical root canal preparation size and taper were standardized in the present study (40/0.06). Standard chemomechanical preparation (40/0.06) significantly reduced the total number of bacteria and *Streptococcus* sp in root canals, which can be explained by the fact that larger preparations permit for a larger volume of irrigant in the canal, increasing the chances for improved chemical effects. One limitation of our study is that we did not have a long period of follow-up for the cases to evaluate the impact of the tested activation methods’ used on the clinical success of the root canal treatment. Other clinical studies are therefore required to correlate the antibacterial efficacy of irrigant activation with its periapical tissue healing capability. Another limitation is the inclusion of teeth with straight root canal anatomy (< 20°), therefore further studies evaluating the efficacy of irrigation activation systems in curved root canals are required.

Bacterial reduction after root canal preparation can be assessed by culture and molecular detection methods. The ddPCR approach was used in this study, because the culture method has low sensitivity, misidentification of cultivable strains with ambiguous phenotype, difficulties in detecting culture-difficult species, and inability to grow many oral species under laboratory artificial conditions [49]. The main advantages of this method are that (i) it has increased precision and robustness and sensitivity in detecting low target copies; (ii) it is relatively insensitive to potential PCR inhibitors; (iii) it measures the absolute number of microRNA copies per microliter of reaction; and (iv) it can show superior diagnostic performance in comparison to a conventional RT-qPCR [50]. Thus, a more accurate evaluation of the antibacterial treatment effectiveness is expected. However, it has the limitation of detecting not only viable but also dead bacteria [51]. Our findings corroborate previous data that showed that DNA may persist in infected teeth even after bacterial death [52]. Considering the results obtained for the total bacteria copy number, the amounts of dead bacteria that remained after chemomechanical preparation appeared to be very small.

## Conclusions

Supplementary steps consisting of SWEEPS activation and PUI promoted further decrease in the bacterial bioburden to levels below those achieved by the chemomechanical procedures alone. SWEEPS activation and PUI were equivalent in terms of reduction in root canal bacteria including *Streptococcus* species and reduction of inflammation detected by IL-1β. Because the long-term outcome of root canal treatment is dependent upon maximal bacterial reduction and inflammation control, the present results are of clinical relevance.

## Data Availability

The data applied in this study are reasonably available from the corresponding author.
